# The Challenge of External Generalisability: Insights from the Bicentric Validation of a [^68^Ga]Ga-PSMA-11 PET Based Radiomics Signature for Primary Prostate Cancer Characterisation Using Histopathology as Reference

**DOI:** 10.3390/cancers16234103

**Published:** 2024-12-07

**Authors:** Samuele Ghezzo, Praveen Gurunath Bharathi, Heying Duan, Paola Mapelli, Philipp Sorgo, Guido Alejandro Davidzon, Carolina Bezzi, Benjamin Inbeh Chung, Ana Maria Samanes Gajate, Alan Eih Chih Thong, Tommaso Russo, Giorgio Brembilla, Andreas Markus Loening, Pejman Ghanouni, Anna Grattagliano, Alberto Briganti, Francesco De Cobelli, Geoffrey Sonn, Arturo Chiti, Andrei Iagaru, Farshad Moradi, Maria Picchio

**Affiliations:** 1 Faculty of Medicine and Surgery, Vita-Salute San Raffaele University, 20132 Milan, Italy; 2 Nuclear Medicine Department, IRCCS San Raffaele Scientific Institute, 20132 Milan, Italy; 3 Division of Nuclear Medicine and Molecular Imaging, Department of Radiology, Stanford University, Stanford, CA 94305, USAfmoradi@stanford.edu (F.M.); 4 Department of Urology, Stanford University, Stanford, CA 94305, USA; 5 Department of Radiology, IRCCS San Raffaele Scientific Institute, 20132 Milan, Italy; 6 Division of Body MRI, Department of Radiology, Stanford University, Stanford, CA 94305, USA; 7 Division of Experimental Oncology, Department of Urology, Urological Research Institute (URI), IRCCS San Raffaele Scientific Institute, 20132 Milan, Italy

**Keywords:** radiomics, PSMA, machine learning, external validation, prostate cancer

## Abstract

**Background**: PSMA PET radiomics is a promising tool for primary prostate cancer (PCa) characterisation. However, small single-centre studies and lack of external validation hinder definitive conclusions on the potential of PSMA PET radiomics in the initial workup of PCa. We aimed to validate a radiomics signature in a larger internal cohort and in an external cohort from a separate centre. **Methods**: One hundred and twenty-seven PCa patients were retrospectively enrolled across two independent hospitals. The first centre (IRCCS San Raffaele Scientific Institute, Centre 1) contributed 62 [^68^Ga]Ga-PSMA-11 PET scans, 20 patients classified as low-grade (ISUP grade < 4), and 42 as high-grade (ISUP grade ≥ 4). The second centre (Stanford University Hospital, Centre 2) provided 65 [^68^Ga]Ga-PSMA-11 PET scans, and 49 low-grade and 16 high-grade patients. A radiomics model previously generated in Centre 1 was tested on the two cohorts separately and afterward on the entire dataset. Then, we evaluated whether the radiomics features selected in the previous investigation could generalise to new data. Several machine learning (ML) models underwent training and testing using 100-fold Monte Carlo cross-validation, independently at both Centre 1 and Centre 2, with a 70–30% train–test split. Additionally, models were trained in one centre and tested in the other, and vice versa. Furthermore, data from both centres were combined for training and testing using Monte Carlo cross-validation. Finally, a new radiomics signature built on this bicentric dataset was proposed. Several performance metrics were computed. **Results:** The previously generated radiomics signature resulted in an area under the receiver operating characteristic curve (AUC) of 80.4% when tested on Centre 1, while it generalised poorly to Centre 2, where it reached an AUC of 62.7%. When the whole cohort was considered, AUC was 72.5%. Similarly, new ML models trained on the previously selected features yielded, at best, an AUC of 80.9% for Centre 1 and performed at chance for Centre 2 (AUC of 49.3%). A new signature built on this bicentric dataset reached, at best, an average AUC of 91.4% in the test set. **Conclusions:** The satisfying performance of radiomics models when used in the original development settings, paired with the poor performance otherwise observed, emphasises the need to consider centre-specific factors and dataset characteristics when developing radiomics models. Combining radiomics datasets is a viable strategy to reduce such centre-specific biases, but external validation is still needed.

## Simple Summary

Despite the promising results of radiomics for the characterisation of primary prostate cancer, its use in clinical practice is still limited due to a lack of results validation. In this study, we validated a previously published radiomics signature in a bicentric setting. Then, we also explored the role of the previously identified radiomics features if models are trained on new data. Furthermore, we investigated the performance of radiomics analysis if feature selection and model training are performed in the bicentric cohort. Results show that radiomics models work well when tested in their development settings, but generalise poorly to new datasets, thus warning about the need to interpret with caution results from single-centre studies or studies lacking proper external validation.

## 1. Introduction

Prostate cancer (PCa) risk stratification is critical for selecting the most effective treatment approach, significantly impacting patient prognosis [1]. Currently, it relies on prostate specific antigen (PSA) values, the clinical stage, and the International Society of Urological Pathology (ISUP) grade obtained from prostate biopsy. However, this approach for PCa characterisation holds several limitations, as biomarkers such as PSA are known to be affected by clinical factors [2] and prostate biopsy does not provide a whole characterisation of the entire prostate or the tumour itself [3]. Systematic template-based biopsies can entirely miss small lesions and under-sample larger ones, resulting in underestimation or overestimation of the actual Gleason score. While targeted biopsies improve lesion sampling, they do not fully overcome these challenges. Moreover, the procedure’s side effects, especially with an increasing number of biopsy cores or repeated biopsies due to inadequate sampling, must be carefully considered [1].

Positron emission tomography (PET) with prostate specific membrane antigen (PSMA) has been widely studied for its utility in characterising primary PCa [4,5]. Evidence supporting the role of this imaging modality in the primary staging and risk assessment of intermediate-to-high-risk PCa patients continues to grow, with studies highlighting its predictive value in assessing lymph node status [6]. However, despite the advantages provided by molecular imaging, core-needle biopsy still represents the tool of choice for primary PCa diagnosis, with non-invasive imaging being used for lesion identification or for biopsy guidance [7,8].

Radiomics, which involves the high-throughput extraction of quantitative features from medical images, has the potential to characterise tumour heterogeneity by revealing patterns invisible to the human eye [9]. PSMA PET radiomics has recently emerged as a promising, non-invasive tool for primary PCa characterisation, with performance at least comparable to biopsy [10,11,12,13,14]. These advancements hint at a future where artificial intelligence (AI)-powered tools will gradually integrate into current diagnostic and management practices for PCa, augmenting standard procedures with supplementary insights.

In recent years, significant efforts have been made to standardise various aspects of radiomics research [15,16,17], including feature definition [18], study workflow [19,20], reporting protocols [20,21], and metrics for evaluating study quality and reliability [22,23]. Additionally, there is a growing recognition among physicians of both the potential benefits and current limitations of this emerging field [24].

Despite this progress and the promising results of PSMA PET radiomics in PCa characterisation, most studies to date remain monocentric, lacking external validation [10,11,12,13]. Single-centre studies restrict the exploration of the impact of centre-specific patient populations and contextual factors on model performance. Moreover, radiomics features are highly sensitive to variations in acquisition protocols and image processing techniques [16,25], complicating the generalisation of radiomics models across different settings. Although various initiatives have been made to harmonise features across scanners and centres [26,27,28,29], differences in patient populations remain an open challenge, potentially hindering the broader applicability of radiomics-based approaches in PCa diagnosis and management.

There has been a notable proliferation of studies introducing novel radiomics signatures [30], yet little attention has been devoted to validating existing ones. The validation of these signatures necessitates a multicentric setting to encompass diverse patient populations and contextual factors. Our primary aim (aim 1) was to validate a radiomics signature previously proposed by the IRCCS San Raffaele Scientific Institute group [10], on a larger internal cohort and an independent external cohort. Secondary aims were to evaluate whether the radiomics features identified in the preliminary investigation can be used to train new machine learning (ML) models, also exploring the impact of centre-specific factors on model performance (aim 2), to assess the potential of radiomics if both feature selection and model training was performed on the bicentric cohort (aim 3), and to compare the performance of radiomics models with maximum Standardised Uptake Value (SUVmax) and biopsy (aim 4).

## 2. Materials and Methods

### 2.1. Patients

**Figure 1:**
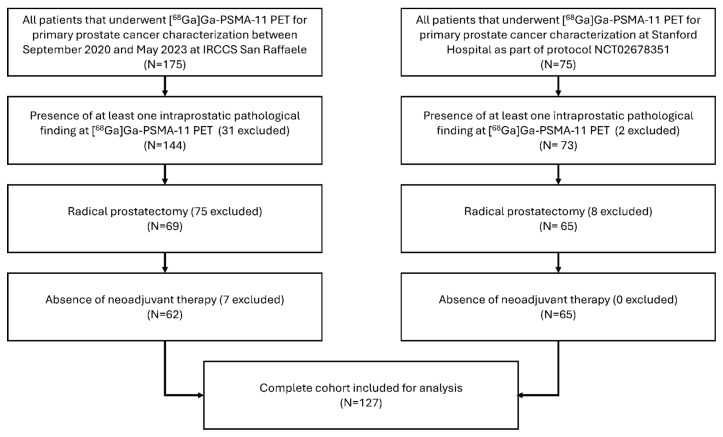
Patients’ selection process.

This bicentric, retrospective study included PCa patients who underwent [^68^Ga]Ga-PSMA-11 PET for primary PCa characterisation prior to planned radical prostatectomy. Specifically, all patients that performed [^68^Ga]Ga-PSMA-11 PET for staging purposes at IRCCS San Raffaele Scientific Institute (Centre 1) between September 2020 and May 2023, and at Stanford University Hospital (Centre 2) between April 2016 and December 2020, as part of the protocol NCT02678351, were initially considered for inclusion. Then, the patients were selected according to the following inclusion criteria: (1) presence of at least one intraprostatic pathological finding on [^68^Ga]Ga-PSMA-11 PET; (2) subsequent radical prostatectomy (RP); and (3) no neoadjuvant treatments. Post-surgical ISUP grade (_PS_ISUP grade) was used to stratify the cohort into low-medium grade (_PS_ISUP grade < 4) and high grade (_PS_ISUP grade ≥ 4). The patients’ selection process is shown in Figure 1. Approval for this retrospective study was obtained from the Institutional Ethics Committees of IRCCS San Raffaele Scientific Institute (101/INT/2022) and Stanford University (IRB-67350). Informed consent was waived due to the retrospective nature of the study.

### 2.2. PET Acquisition Protocols

At Centre 1, PET scans were acquired using either a Signa PET/MRI 3 Tesla system (N = 39) or Discovery-STE or Discovery-690 PET/CT (N = 23) (all GE Healthcare, Waukesha, WI, USA). At Centre 2, all PET images were acquired using the Signa PET/MRI 3 Tesla system (GE Healthcare, Waukesha, WI, USA). At both centres, [^68^Ga]Ga-PSMA-11 PET scans were acquired according to the standard clinical practice, following the joint EANM and SNMMI procedure guidelines for PCa imaging [31]. Specifically, [^68^Ga]Ga-PSMA-11 PET/CT and PET/MRI scans began approximately 60 min after the injection of approximately 2 MBq [^68^Ga]Ga-PSMA-11 per kilogram body weight, with a duration of 4 min per bed position.

Interpretation of [^68^Ga]Ga-PSMA-11 PET images was conducted by two nuclear medicine physicians using the Advantage Workstation (AW, GE Healthcare, Waukesha, WI, USA), both at Centre 1 and Centre 2. Positive findings for malignancy were determined by the presence of intraprostatic focal and intense uptake of [^68^Ga]Ga-PSMA-11 not attributable to physiological and/or not specific uptake, either with or without corresponding alterations visible on the co-registered CT or MR images, as routinely done in clinical practice.

### 2.3. Volume of Interest Segmentation

The whole prostate was used as the volume of interest (VOI) as the scope of this study was to validate the radiomics signature identified in a preliminary investigation using the whole prostate as VOI [10]. Moreover, extracting radiomic features (RFs) from the entire prostate provides a more comprehensive assessment of tumour heterogeneity, while also avoiding sampling errors, overcoming the limitations of radiomics for small lesions (fewer than 64 voxels) [16], and the challenges associated with multi-lesion characterisation.

Specifically, at both centres, the whole prostate was manually segmented slice by slice on co-registered PET/CT or PET/MR images. The co-registered images were loaded on 3D Slicer, version 5.1.0 [32], and were visualised on the axial plane. PET images were visualised with a SUV window ranging from 0 to 5, but the readers could adjust the windowing if needed. CT or MRI was used to define the prostate contour, because of its high anatomical accuracy, while PET images were used to carefully exclude any bladder spill-in activity. Although not used for segmentation, the sagittal and coronal planes could be used to control the quality and consistency of the contouring. PET images were set as the “Master Volume”, and therefore the VOIs were drawn directly on PET. At Centre 1, segmentations were made by a pool of 2 radiologists (T.R. and G.B.), while at Centre 2 this was performed by a pool of 2 nuclear medicine physicians (H.D. and P.S.).

### 2.4. Radiomics Feature Extraction and Harmonisation

PET images were converted to SUV body weight values, then VOIs were normalised, discretised using fixed bin width (FBW = 0.3), and then resampled to 2.0 × 2.0 × 2.0 mm^3^ voxels using B-spline interpolation on PyRadiomics [33], as done in [10]. Afterwards, 102 3D Image Biomarker Standardisation Initiative (IBSI)-compliant RFs were extracted from the resampled VOIs on PET images using PyRadiomics, version 3.0.1; see Supplementary Table S1 for the complete feature extraction configuration. SUVmax was also computed for each VOI. When analysing data from patients at Centre 1, we used the ComBat software (https://forlhac.shinyapps.io/Shiny_ComBat/, accessed on 18 April 2024) to harmonise radiomic features extracted from images acquired using different scanners. For experiments involving only Centre 2 data, harmonisation was unnecessary since all patients were imaged with the same scanner. However, when combining data from both centres, a two-step harmonisation process was applied: first, scanner differences within Centre 1 were addressed, followed by harmonisation of radiomic features across the two centres.

### 2.5. Radiomics Signature Validation

The [^68^Ga]Ga-PSMA-11 PET radiomics signature presented in [10], for predicting _PS_ISUP grade ≥ 4, consists of a support vector machine with C = 2.5, gamma = scale, and kernel = linear, using Zone Entropy, extracted from the grey level size zone matrix (GLSZM) and Least Axis Length (shape) as input, that was previously trained on 47 patients examined at Centre 1. To assess its generalisation ability, this signature was tested on sixty-two patients examined at Centre 1. Then, the same fully trained model was tested on sixty-five patients investigated at Centre 2, and finally, its performance was evaluated on the entire cohort, comprising 127 patients (all patients examined at Centre 1 and Centre 2).

### 2.6. Models Training Using Previously Selected Radiomics Features

To mitigate potential biases from various sources—such as a small sample size in signature development, class imbalance, centre-specific factors, differences between training and validation sets, and similarities or differences between the cohorts used for signature identification and validation—the investigation of the role of previously selected radiomics features in training new ML models was designed as follows:

As for aim 1, features were selected based on a preliminary investigation made at Centre 1 [10], and two RFs were included for analyses, namely Zone Entropy (GLSZM) and Least Axis Length (shape).

**Figure 2:**
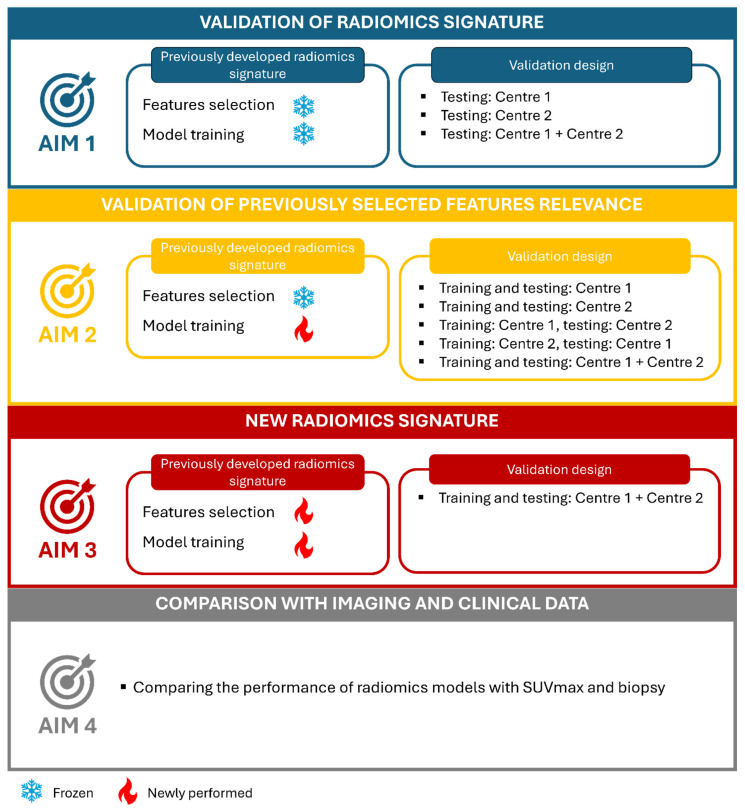
Study design.

Then, a comprehensive ensemble of ML models including a random forest classifier, support vector machine (SVM), logistic regression (LR), and a meta-model yielding the majority vote from these classifiers was trained and evaluated for _PS_ISUP grade prediction. To assess the impact of differences in training and testing sets, models were (1) trained and tested on Centre 1, (2) trained and tested on Centre 2, (3) trained on Centre 1 and tested on Centre 2, (4) trained on Centre 2 and tested on Centre 1, and (5) trained and tested on both Centre 1 and Centre 2. Figure 2 summarises the study design.

Except for experiments where one centre was used for training and the other one for testing, a Monte Carlo cross-validation technique, iterated 100 times, was employed for both model training and assessment. During each iteration, 70% of the stratified dataset was allocated for training purposes while the remaining 30% was reserved for testing. The final estimate of model performance was derived from the average results across the test sets.

**Figure 3:**
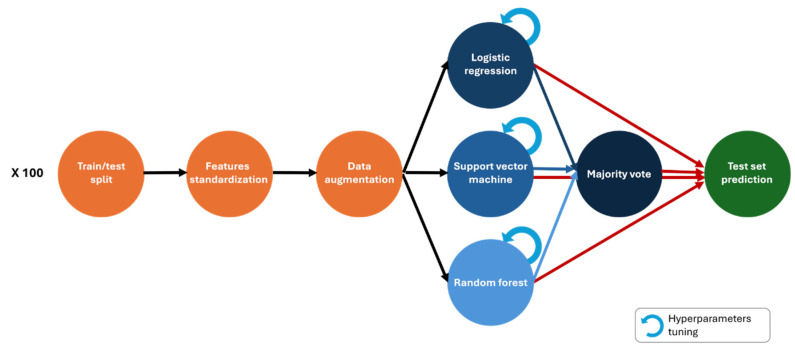
Machine learning workflow.

Radiomics features were standardised using the z-score in the training set, and then mean and standard deviation were fitted to the test set, and the Synthetic Minority Over-sampling Technique (SMOTE) was implemented to rebalance the training features, ensuring a 1:1 ratio with the prevailing class. Subsequently, models were exclusively evaluated on non-augmented cohorts. Hyperparameter optimisation was conducted using five-fold stratified cross-validation within the training sets, employing grid searches detailed in Supplementary Table S2. Consequently, when Monte Carlo cross-validation was used, each iteration entailed the selection and utilisation of distinct hyperparameter sets for model development. The weak learners contributing to the meta-model were consistently trained with the optimal hyperparameters identified through prior grid search exploration. Hyperparameter tuning was based on the area under the receiver operating characteristic curve (AUC) in the training set. The complete pipeline for model development is depicted in Figure 3.

### 2.7. New Radiomics Signature Generation

To explore the potential of radiomics analysis in this bicentric cohort, a new radiomics signature was developed by performing feature selection and model training afresh. Data from the two centres were combined, and features with a high correlation (Spearman coefficient ≥ 0.8) were filtered by randomly dropping one of the correlated pairs. Least absolute shrinkage and selection operator (LASSO) regression was then applied to select relevant radiomic features from the training set. ML models were trained and tested using Monte Carlo cross-validation, as previously described, with 70% of the dataset allocated for training and 30% for testing in each iteration. The final performance of each radiomics model was determined by averaging the performance of the ML models across 100 iterations.

### 2.8. Comparison with Imaging and Clinical Data

Receiver operating characteristic (ROC) analysis was used to investigate the predictive role of SUVmax for the prediction of _PS_ISUP grade ≥ 4, and AUC values, along with their 95% confidence interval (CI), were computed. Optimal cut-off was derived by choosing the value corresponding to the point on the ROC curve nearest to the upper left corner of the ROC graph (Youden Index method). Sensitivity (SN), specificity (SP), positive predictive value (PPV), and negative predictive value (NPV) of SUVmax on Centre 1, Centre 2, and the entire dataset was reported. Finally, the performance of histological examination of prostate biopsy cores was assessed on Centre 1, Centre 2, and the whole dataset, and was compared to that of the radiomics models.

### 2.9. Statistical Analysis

Continuous variables were reported as median and interquartile range (IQR) and compared by means of the Mann–Whitney U test. Discrete variables were summarised as counts and percentages, and comparisons were conducted using the Fisher exact test. Evaluation of radiomics model performance on the test sets encompassed AUC, along with 95% CI, SN, SP, PPV, and NPV. Given that results from Monte Carlo cross-validation represent averages across 100 iterations, corresponding standard deviations are also provided for comprehensive analysis.

AUCs of the previously trained ML model tested on Centre 1, Centre 2, and the entire dataset were compared using the De Long test. The AUC distributions of the newly trained radiomics models, generated with random forest, support vector machine, and logistic regression, and their aggregation via majority vote, were compared using the Kruskal–Wallis test. To better investigate the differences among the generated models, post hoc comparisons were conducted using the Wilcoxon-signed rank test. Conversely, in those experiments where only one AUC value for model is present (i.e., when one centre is used for training and the other one for testing), the De Long test was employed to compare the AUCs of the radiomics models.

The increased likelihood of type I errors from familywise error rates was corrected using the false discovery rate. *p*-values below 0.05 were deemed statistically significant. Statistical analyses were performed with R statistical software (version 4.0.5) [34], and ML models were generated with Python (version 3.8.3, Python Software Foundation, Wilmington, DE, USA).

### 2.10. Radiomics Study Quality Assessment

The CheckList for Evaluation of Radiomics research (CLEAR checklist) [20,35] was used to ensure readability, transparency, and the presence of comprehensive information required to reproduce our findings, and the METhodological RadiomICs Score (METRICS) [23] was used to assess the methodological quality of our research.

## 3. Results

### 3.1. Patients

In the present study, a total of 127 patients were enrolled. Centre 1 included 62 patients with a median age of 65 years (IQR: 60–70 years) and a median initial prostate-specific antigen (iPSA) level of 6.53 ng/mL (IQR: 4.91–7.89 ng/mL). Centre 2 included 65 patients, with a median age of 66 years (IQR: 60-68 years) and a median iPSA level of 8 ng/mL (IQR: 5.4–11 ng/mL). Notably, 42 patients from Centre 1 had a _PS_ISUP grade ≥ 4 (67.7%) while in Centre 2, only 16 patients exhibited high-grade disease at prostatectomy (*p* < 0.001). The patients’ characteristics are reported in Table 1.

### 3.2. Radiomics Signature Validation

The radiomics signature previously generated at Centre 1 performed best on the enlarged cohort examined at Centre 1, reaching an AUC of 0.804 (95% CI: 0.686–0.896), while it generalised poorly to Centre 2, where it yielded an AUC of 0.628 (95% CI: 0.502–0.747). This previously trained model resulted in an AUC of 0.725 (95% CI: 0.638–0.799) when used on the entire cohort, encompassing patients enrolled at both Centre 1 and Centre 2. However, the difference in model performance across cohorts was not statistically significant (*p* > 0.05), probably due to the large confidence intervals; see Table 2 for detailed information on model performance across the two centres. The previously generated radiomics model, first presented in [10] and validated in this paper, is made available on GitHub, https://github.com/samueleghezzo/bicentric_validation_radiomics_signature (accessed on 4 October 2024).

### 3.3. Models Trained Using Previously Selected Radiomics Features

**Figure 4:**
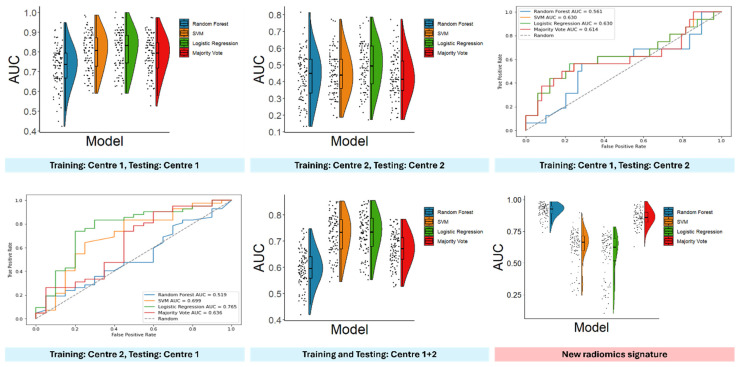
AUC of the machine learning models developed in this study.

The description of ML models after hyperparameter optimisation is reported in Supplementary Table S3, while the performance of the best models, measured with AUC, SN, SP, PPV, and NPV, is reported in Table 3 and Figure 4 (detailed information on all the generated models is provided in Supplementary Table S4).

The Kruskal–Wallis test highlighted different performances for the different classifiers (*p* value: <0.001, 0.004, <0.001), and post hoc comparisons using the Wilcoxon-signed rank test highlighted the inferior performance of random forest and majority vote, compared to LR and SVM (Table 3). The De Long test showed a better performance for LR and SVM also when data from Centre 2 were used for training and data from Centre 1 were used for testing, while no differences were found in the performance of the investigated classifiers when Centre 1 was used for training and Centre 2 for testing (Supplementary Table S5).

When training and testing was performed at Centre 1, LR reached an average AUC of 0.809 (95% CI: 0.785–0.833). However, when the ML models were trained and tested at Centre 2, the best average AUC for all four ML models was 0.493 (95% CI: 0.461–0.525). When ML models were trained at Centre 1 and tested on data acquired at Centre 2, SVM reached the highest AUC of 0.630 (95% CI: 0.509–0.738). When training was performed at Centre 2, and testing was conducted on data gathered at Centre 1, LR yielded an AUC of 0.765 (95% CI: 0.638–0.847). Finally, when data from both centres were used for both training and testing, LR reached an average AUC of 0.728 (95% CI: 0.715–0.741). The aggregation of models’ predictions via majority vote did not improve their performance and there were no signs of overfitting due to hyperparameter tuning (Table 3 and Supplementary Tables S4 and S5).

### 3.4. New Radiomics Signature

The four radiomics features most selected were glcm_ClusterProminence, firstorder_Energy, shape_VoxelVolume, and glszm_LargeAreaEmphasis, used in 98, 65, 59, and 58 iterations, respectively. The fifth most selected feature was glrlm_RunLengthNonUniformity, selected only 12 times (see Supplementary Figure S1 for a graphical representation of the selected features across the 100 iterations of Monte Carlo cross-validation). On average, the best classifier was the random forest classifier, yielding an average AUC of 0.914 in the test set (95% CI: 0.850–0.956), while the worst one was LR, reaching an average AUC = 0.565 in the test set (95%CI: 0.476–0.655). The Kruskal–Wallis test highlighted different performances among the used classifiers (*p* < 0.001), which were also confirmed by post hoc comparisons (see Supplementary Table S5). All metrics showing the performance of the generated ML models using the newly selected radiomics features are shown in Table 4 and Figure 4 (Supplementary Table S3 for hyperparameter settings).

A random forest classifier trained on the full sample using the four most selected radiomics features in the 100 iterations of Monte Carlo cross-validation is available on GitHub, https://github.com/samueleghezzo/bicentric_validation_radiomics_signature (accessed on 4 October 2024). This study has a METRICS score of 85.3% (excellent). A full report on METRICS and the CLEAR checklist for this study can be found in Supplementary Tables S6 and S7.

### 3.5. Comparison with Imaging and Clinical Data

**Figure 5:**
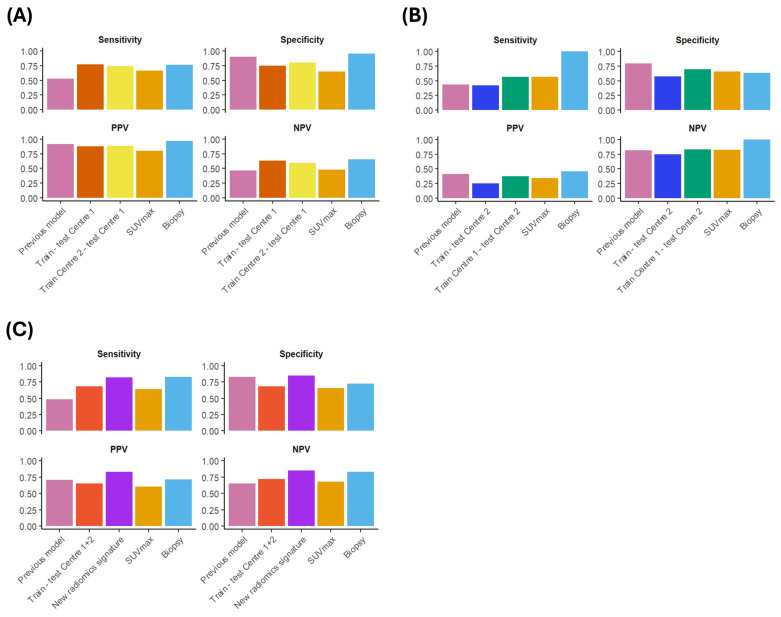
Comparison between radiomics models, SUVmax, and biopsy. (**A**) Performance of radiomics models, SUVmax, and biopsy when tested at Centre 1. (**B**) Performance of radiomics models, SUVmax, and biopsy when tested at Centre 2. (**C**) Performance of radiomics models, SUVmax, and biopsy when tested on the entire cohort (Centre 1 + Centre 2).

At Centre 1, SUVmax resulted in an AUC of 0.688 (95% CI: 0.552–0.826), and considering an optimal cut-off of 14.185 to classify patients as high-grade, SN was 0.667, SP = 0.650, PPV = 0.800, and NPV = 0.481. At Centre 2, SUVmax yielded an AUC of 0.556 (95% CI: 0.388–0.724), and using an optimal cut-off of 14.085 to classify patients as high-grade, SN was 0.563, SP = 0.653, PPV = 0.346, and NPV = 0.821. Combining data gathered at Centre 1 and Centre 2, SUVmax reached an AUC of 0.656 (95% CI: 0.560–0.752) with an optimal threshold of 14.185, resulting in SN = 0.638, SP = 0.652, PPV = 0.607, and NPV = 0.681. See Figure 5 for the comparison between radiomics models and SUVmax.

At Centre 1, of the 62 patients investigated, 29 were classified as low-grade ISUP (ISUP < 4) based on biopsy results, while the remaining 33 were categorised as high-grade. Post-surgical histopathological analysis confirmed low-grade ISUP in 19 out of 29 patients and high-grade ISUP in 32 out of 33 patients. Thus, biopsy had an SN of 0.762, SP = 0.950, PPV = 0.969, and NPV = 0.655 at Centre 1. At Centre 2, 31/65 patients were classified as low-grade ISUP while 33/65 showed high-grade ISUP at biopsy. Biopsy results were unavailable for one participant. Post-prostatectomy histopathology showed low-grade ISUP in 49/65 and high-grade ISUP in 16/65 patients, resulting in an SN of 1, SP = 0.633, PPV = 0.455, and NPV = 1 at Centre 2. Overall, combining the cohorts examined at Centre 1 and Centre 2, biopsy showed an SN of 0.824, SP = 0.724, PPV = 0.712, and NPV = 0.833. See Figure 5 for the comparison between radiomics models and biopsy.

## 4. Discussion

This study reports a retrospective analysis evaluating the generalisation ability of a radiomics signature derived from [^68^Ga]Ga-PSMA-11 PET imaging [10]. Data from two centres were integrated, and a radiomics signature previously generated at Centre 1 was tested on a larger internal cohort and on an independent external dataset from Centre 2. Results showed that the radiomics model worked well when tested in the same development settings, but generalised poorly to another centre.

Several retrospective, single-centre studies have demonstrated the promising potential of PSMA PET radiomics for the preoperative characterisation of primary PCa. Zamboglou and colleagues achieved an AUC of 0.84 in distinguishing between ISUP ≤ 3 and ISUP ≥ 4 [13]. Papp et al. developed a complex ensemble of random forest classifiers, incorporating features from PSMA PET and MRI, which yielded an AUC of 0.86 for predicting ISUP grade [12]. Similarly, Cysouw constructed [^18^F]F-DCFPyL PET radiomics models for predicting ISUP grade, lymph node involvement, and extracapsular extension [36].

In a recent study, Luining validated Cysouw’s radiomics signatures with an independent cohort of 51 patients (24 from the same centre where the signatures were generated, and 27 from a different institution). This validation revealed a significant drop in model performance for predicting lymph node involvement and extracapsular extension, with the AUCs falling from 0.86 and 0.76 in the original study to 0.57 and 0.63 in the independent cohort, respectively. The model predicting ISUP grade remained relatively stable, with the AUC decreasing from 0.81 to 0.78 [37]. However, in the latter study, the performance of the radiomics model was tested only in the combined independent cohort. This approach did not evaluate the models’ performance in each centre separately or account for differences in cohorts, imaging protocols, and other contextual factors that could have influenced the models’ efficacy.

In contrast, our study assessed the performance of the previously generated radiomics signature across different settings. This allowed us to discover that the discrete performance of the radiomics model in the bicentric dataset was driven by the good stability of the signature when applied to the larger internal cohort, paired with a poor performance on the external dataset.

There are several factors that could have contributed to the lack of generalisability of the previously published radiomics signature to a second centre. First, even though PET images were acquired following the joint EANM and SNMMI procedure guidelines for PCa imaging at both institutions [31], the differences in acquisition protocols and image reconstruction could have introduced noise. Furthermore, readers at both centres followed the same instructions for VOI delineation, but since it was not possible to share the images across hospitals, it was not feasible to test, and adjust for, inter-reader variability across institutions. The patients’ demographics and disease characteristics were significantly different across the two centres, resembling real-world evidence. Finally, dataset- and centre-specific information can inadvertently creep into the model during the model training process, particularly for small datasets, resulting in artificially high performance in the validation subset despite suboptimal generalisability for completely different datasets.

Furthermore, since the original radiomics model was developed based on only 47 patients, and various sources of bias could have affected the model’s performance, we assessed whether at least the RFs selected in that investigation had the potential to generalise to new data. To do so, we used the two previously identified RFs to train new ML models, investigating in depth the impact of imaging protocols, patient characteristics, disparities between training and testing sets, and class imbalance on the models’ performance. This analysis showed that the step of feature selection is also largely dependent on the dataset used to develop the radiomics signature. Indeed, the highest performances were observed when the ML models were tested on Centre 1, while, when Centre 2 was used for testing, the models performed randomly. This may be due to the difference in class distribution between the two centres, inter-reader variability in the process of VOI segmentation, variations in the acquisition protocols, or differences in the disease characteristics across the two centres.

Finally, we developed a new radiomics signature using the entire cohort investigated in this study to assess if radiomics had the potential to predict _PS_ISUP grade in this bicentric cohort, or if the differences between patients examined at Centre 1 and Centre 2 were too large, hampering the possibility to produce meaningful models. The new radiomics signature had a performance comparable to that of biopsy, and similar to that reported in the previous investigation conducted at Centre 1 [10]. These findings emphasise the importance of considering centre-specific factors, such as imaging protocols and patient populations, when conducting radiomics analysis. However, in the absence of an independent test set acquired at a third centre, and considering the previous findings, it is not possible to draw conclusions on the potential generalisation ability of this newly proposed radiomics signature. For this reason, we are making the trained bicentric signature available online for the larger community to test it.

Notably, variability was observed not only in the performance of radiomics models, but also in biopsy results. At Centre 1, biopsies demonstrated high specificity but limited sensitivity, whereas at Centre 2, they showed the opposite pattern, with a sensitivity of 1 and a specificity of 0.633. SUVmax seemed to have a limited potential to stratify patients to low and high ISUP grade in both cohorts, thus, highlighting the need for more effective strategies to accurately grade PCa patients.

For radiomics to be useful in clinical practice, and to allow scalability, there is an urgent need for large, prospective, multicentric studies with harmonised data acquisition, interpretation, and labelling procedures. One alternative is the generation of centre-specific radiomics models, where each institution uses models calibrated to its specific scanners, patient demographics, clinical characteristics, and imaging procedures. Even in this case, extensive and prospective validation of the models would still be necessary. Another solution involves deploying federated learning approaches [38,39]. Federated learning enables collaborative model training across multiple centres while preserving data privacy and security, thereby facilitating the development of more robust and generalisable predictive models for PCa risk stratification.

One notable strength of the study lies in its methodological rigor, as evidenced using guidelines for radiomics in nuclear medicine [19] and the CLEAR checklist [20] during study design, conduction, and reporting. By employing a bicentric approach, the study not only increased in sample size, but also enhanced the external validity of the results, allowing conclusions to be drawn regarding the reproducibility of radiomics signatures across different clinical settings [40]. The study’s methodological quality assessment, as evaluated through METRICS [23], further strengthens its credibility and transparency. Furthermore, since we observed that the radiomics signatures worked well in their development settings but generalised poorly to new data, we are making two fully trained models freely available online. This allows the broader community to test them on their own data, offering valuable insights into their generalisability.

A potential limitation of this work lies in the inherent heterogeneity across different centres in terms of patient demographics, disease characteristics, and population distribution based on ISUP grades. Several efforts have been made in recent years to harmonise RFs across scanners and centres [26,27,28,29]. However, addressing discrepancies in patient populations remains a challenge that requires models trained on large and diverse datasets. Another limitation resides in the relatively low sample size. Although data were collected from two institutions, creating a cohort larger than those investigated in most previous studies in this setting [11,12,13,36], a sample size of 127 patients might still be small for ML modelling. Although the new, bicentric, radiomics signature was developed to control for the potential utility of radiomics when both feature selection and model training was performed on the bicentric cohort, the inclusion of a third, independent centre would have offered deeper insights into the generalisability of a [^68^Ga]Ga-PSMA-11 PET-based radiomics signature derived from a larger and more heterogeneous dataset. Without an independent test set it is not possible to draw conclusions on its generalisability. However, the fully trained bicentric model has been made freely available online, enabling the radiomics community to test it on their own data, potentially providing further insights.

Variations in patient populations and disease profiles between centres may influence the performance of ML models trained on data from one centre when tested or validated on data from another centre. To mitigate this limitation and enhance the robustness of predictive models across diverse clinical settings, we are working on deploying federated learning approaches [38,39]. Federated learning enables collaborative model training across multiple centres while preserving data privacy and security, thus facilitating the development of more robust and generalisable predictive models for prostate cancer risk stratification. By leveraging federated learning, future studies aim to overcome the challenges associated with centre-specific variations and enhance the reliability and scalability of radiomics-based predictive models in clinical practice.

## 5. Conclusions

Our work provided valuable insights into the generalisability of radiomics analysis using [^68^Ga]Ga-PSMA-11 PET imaging to predict ISUP grades in PCa patients. The radiomics signature previously presented by Centre 1 failed to generalise to data from Centre 2, as did the radiomics features selected in that earlier study. Our findings show that while [^68^Ga]Ga-PSMA-11 PET radiomics models achieve satisfying performance in their original development settings, they seem to struggle to generalise to new datasets, thus limiting their scalability and clinical transferability. This has critical implications for clinical practice, as accurate predictions are essential for patient stratification, treatment planning, and prognosis. By employing a rigorous methodology and comprehensive validation approach, we highlight the necessity of considering centre-specific factors and dataset characteristics in hand-crafted radiomics analysis. Furthermore, our findings warn against the overly optimistic results frequently reported in single-centre studies or studies lacking proper external validation.

## Figures and Tables

**Table 1:** Patients’ characteristics.

		Centre 1	Centre 2	*p*-Value
Age (years), median (IQR)		65 (60–70)	66 (60–68)	
iPSA (ng/mL), median (IQR)		6.53 (4.91–7.89)	8 (5.4–11)	
ISUP grade (biopsy), n (%)				
	1	2 (3.22)	1 (1.54)	0.64
	2	8 (12.90)	12 (18.46)	
	3	19 (30.65)	18 (27.69)	
	4	20 (32.26)	15 (23.08)	
	5	13 (20.97)	18 (27.69)	
	Not available	0 (0)	1 (1.54)	
ISUP grade (prostatectomy), n (%)				
	1	0 (0)	1 (1.54)	<0.001 ***
	2	7 (11.29)	22 (33.85)	
	3	13 (20.97)	26 (40)	
	4	8 (12.90)	2 (3.07)	
	5	34 (54.84)	14 (21.54)	

*** *p* < 0.001.

**Table 2:** Radiomics signature validation.

Validation Cohort	AUC	SN	SP	PPV	NPV
Centre 1	0.804 (0.686–0.896)	0.524	0.9	0.913	0.462
Centre 2	0.627 (0.502–0.747)	0.438	0.796	0.412	0.813
Centre 1 + Centre 2	0.725 (0.638–0.799)	0.483	0.826	0.707	0.655

95% CI in brackets.

**Table 3:** Best radiomics models with previously selected radiomics features.

Training Cohort	Testing Cohort	Train- AUC	Test- AUC	Test- SN	Test- SP	Test- PPV	Test- NPV
Centre 1	Centre 1	0.819	0.809 (0.785–0.833)	0.768	0.750	0.879	0.631
Centre 2	Centre 2	0.551	0.493 (0.461–0.525)	0.422	0.571	0.250	0.750
Centre 1	Centre 2	0.806	0.630 (0.509–0.738)	0.563	0.694	0.375	0.829
Centre 2	Centre 1	0.542	0.765 (0.638–0.847)	0.738	0.800	0.886	0.593
Centre 1+ Centre 2	Centre 1 + Centre 2	0.706	0.728 (0.715–0.741)	0.683	0.680	0.650	0.721

95% CI in brackets.

**Table 4:** New radiomics signature.

Classifier	Train- AUC	Test- AUC	Test- SN	Test- SP	Test- PPV	Test- NPV
Logistic regression	0.630	0.565 (0.476–0.655)	0.484	0.660	0.553	0.597
Random forest	0.936	0.914 (0.850–0.956)	0.819	0.849	0.832	0.851
Support vector machine	0.641	0.628 (0.539–0.714)	0.482	0.691	0.630	0.558
Majority vote	-	0.861 (0.794–0.920)	0.746	0.829	0.795	0.800

95% CI in brackets.

## Data Availability

The fully trained radiomics models investigated in this study are made available at https://github.com/samueleghezzo/bicentric_validation_radiomics_signature (accessed on 4 October 2024).

## Supplementary Materials

The following supporting information can be downloaded at: https://www.mdpi.com/article/10.3390/cancers16234103/s1, Table S1: Features extraction parameters, Table S2: Hyperparameters investigated; Table S3: Hyperparameters used; Table S4: Performance of newly trained machine learning models with pre-defined radiomics features; Table S5: Comparisons between newly trained machine learning models; Table S6: METRICS score; Table S7: CLEAR checklist; Figure S1: Features used in the new bicentric signature.
