# Supplementary material for: The Challenge of External Generalisability: Insights from the Bicentric Validation of a [68Ga]Ga-PSMA-11 PET Based Radiomics Signature for Primary Prostate Cancer Characterisation Using Histopathology as Reference
Source: Cancers (Basel). 2024 Dec 7;16(23):4103. doi: 10.3390/cancers16234103 (PMC11640655; doi:10.3390/cancers16234103)

Table S1. Features extraction parameters

**setting**	
normalize	True
binWidth	0.3
resampledPixelSpacing	2, 2, 2
label	1
interpolator	sitkBSpline
minimumROISize	64
**imageType**	
Original	{}
**featureClass**	
shape	'MeshVolume', 'VoxelVolume', 'SurfaceArea', 'SurfaceVolumeRatio', 'Sphericity', 'Maximum3DDiameter', 'Maximum2DDiameterSlice', 'Maximum2DDiameterColumn', 'Maximum2DDiameterRow', 'MajorAxisLength', 'MinorAxisLength', 'LeastAxisLength', 'Elongation', 'Flatness'
firstorder	'Energy', 'Entropy','Minimum', '10Percentile', '90Percentile', 'Maximum', 'Mean', 'Range','InterquartileRange', 'Median', 'MeanAbsoluteDeviation', 'RobustMeanAbsoluteDeviation', 'RootMeanSquared', 'Skewness', 'Kurtosis', 'Variance', 'Uniformity'
glcm	'Autocorrelation', 'ClusterProminence', 'ClusterShade', 'ClusterTendency', 'Contrast', 'Correlation', 'DifferenceAverage', 'DifferenceVariance', 'JointEntropy', 'JointEnergy', 'Imc1', 'Imc2', 'Idm', 'MCC', 'Idmn', 'Id', 'Idn', 'InverseVariance', 'MaximumProbability', 'SumEntropy', 'SumSquares'
glszm	'SmallAreaEmphasis', 'LargeAreaEmphasis', 'GrayLevelNonUniformity', 'GrayLevelNonUniformityNormalized', 'SizeZoneNonUniformity', 'SizeZoneNonUniformityNormalized', 'ZonePercentage', 'GrayLevelVariance', 'ZoneVariance', 'ZoneEntropy', 'LowGrayLevelZoneEmphasis', 'HighGrayLevelZoneEmphasis', 'SmallAreaLowGrayLevelEmphasis', 'SmallAreaHighGrayLevelEmphasis', 'LargeAreaLowGrayLevelEmphasis', 'LargeAreaHighGrayLevelEmphasis'
glrlm	'ShortRunEmphasis', 'LongRunEmphasis', 'GrayLevelNonUniformity', 'GrayLevelNonUniformityNormalized', 'RunLengthNonUniformity', 'RunLengthNonUniformityNormalized', 'RunPercentage', 'GrayLevelVariance', 'RunVariance', 'RunEntropy', 'LowGrayLevelRunEmphasis', 'HighGrayLevelRunEmphasis', 'ShortRunLowGrayLevelEmphasis', 'ShortRunHighGrayLevelEmphasis', 'LongRunLowGrayLevelEmphasis', 'LongRunHighGrayLevelEmphasis'

ngtdm	'Coarseness', 'Contrast', 'Busyness', 'Complexity', 'Strength'
gldm	'SmallDependenceEmphasis', 'LargeDependenceEmphasis', 'GrayLevelNonUniformity', 'DependenceNonUniformity', 'GrayLevelVariance', 'DependenceVariance', 'DependenceEntropy', 'LowGrayLevelEmphasis', 'HighGrayLevelEmphasis', 'SmallDependenceLowGrayLevelEmphasis', 'LargeDependenceLowGrayLevelEmphasis', 'SmallDependenceHighGrayLevelEmphasis', 'LargeDependenceHighGrayLevelEmphasis'

Table S2. Hyperparameters investigated

**Random forest**	
n_estimators	25, 50, 75, 100, 125, 150
min_samples_split	2, 3
min_samples_leaf	1, 2
max_features	2

**Support vector machine**	
C	0.5, 1, 1.5, 2, 2.5, 3
Gamma	0.5, 0.8, 1, 1.5
Kernel	Linear
Probability	True

**Logistic regression**	
Solver	'lbfgs', 'newton-cholesky', 'liblinear'
Penalty	L2
C	0.1, 0.5, 0.8, 1, 1.25, 2

Table S3a. hyperparameters used -aim 2

**Logistic regression**			
C	Penalty	Solver	Count
0.1	L2	lbfgs	220
0.1	L2	liblinear	7
0.5	L2	lbfgs	44
0.5	L2	liblinear	3
0.8	L2	lbfgs	9
0.8	L2	liblinear	1
2	L2	lbfgs	8
2	L2	liblinear	2
1	L2	lbfgs	4
1	L2	liblinear	1
1.25	L2	lbfgs	2
1	L2	newton-cholesky	1

**Support vector machine**				
C	Gamma	Kernel	Probability	Count
0.5	0.5	Linear	True	198

1	0.5	Linear	True	51
1.5	0.5	Linear	True	23
2	0.5	Linear	True	15
2.5	0.5	Linear	True	8
3	0.5	Linear	True	7

**Random forest**				
Min sample leaf	Min sample split	No. estimators	Max features	Count
1	2	100	2	15
1	2	125	2	6
1	2	150	2	13
1	2	25	2	30
1	2	50	2	16
1	2	75	2	11
1	3	100	2	5
1	3	125	2	5
1	3	150	2	9
1	3	25	2	20
1	3	50	2	16
1	3	75	2	9
2	2	100	2	10
2	2	125	2	9
2	2	150	2	8
2	2	25	2	20
2	2	50	2	19
2	2	75	2	13
2	3	125	2	8
2	3	150	2	9
2	3	25	2	23
2	3	50	2	10
2	3	75	2	13
2	3	100	2	5

Table S3b. hyperparameters used -Aim 3

**Logistic regression**			
C	Penalty	Solver	Count
0.1	'l2'	'lbfgs'	27
0.1	'l2'	'liblinear'	5
0.1	'l2'	'newton-cg'	6
0.5	'l2'	'lbfgs'	3
0.5	'l2'	'liblinear'	2
0.5	'l2'	'newton-cg'	4
0.8	'l2'	'lbfgs'	3
0.8	'l2'	'liblinear'	2
0.8	'l2'	'newton-cg'	7
1	'l2'	'lbfgs'	3
1	'l2'	'liblinear'	3
1	'l2'	'newton-cg'	3
1.25	'l2'	'lbfgs'	1
1.25	'l2'	'liblinear'	6
1.25	'l2'	'newton-cg'	8

2	'l2'	'lbfgs'	6
2	'l2'	'liblinear'	7
2	'l2'	'newton-cg'	4

**Support vector machine**				
C	Gamma	kernel	Probability	Count
0.5	'auto'	'linear'	True	9
0.5	'scale'	'linear'	True	22
0.5	0.5	'linear'	True	4
0.5	0.8	'linear'	True	4
0.5	1	'linear'	True	2
0.5	1.5	'linear'	True	1
1	'auto'	'linear'	True	2
1	0.5	'linear'	True	3
1	0.8	'linear'	True	2
1	1	'linear'	True	2
1	1.5	'linear'	True	2
1.5	'auto'	'linear'	True	2
1.5	'scale'	'linear'	True	2
1.5	0.5	'linear'	True	1
1.5	0.8	'linear'	True	2
1.5	1	'linear'	True	2
1.5	1.5	'linear'	True	1
2	'auto'	'linear'	True	1
2	'scale'	'linear'	True	3
2	0.5	'linear'	True	2
2	1	'linear'	True	1
2	1.5	'linear'	True	2
2.5	'auto'	'linear'	True	1
2.5	'scale'	'linear'	True	2
2.5	0.5	'linear'	True	2
2.5	0.8	'linear'	True	7
2.5	1.5	'linear'	True	2
3	'auto'	'linear'	True	3
3	'scale'	'linear'	True	3
3	0.5	'linear'	True	1
3	0.8	'linear'	True	3
3	1	'linear'	True	2
3	1.5	'linear'	True	2

**Random forest**				
Max features	Min sample leaf	Min sample split	No. estimators	Count
'log2'	1	2	25	2
'log2'	1	2	50	1
'log2'	1	2	75	1

'log2'	1	2	100	2
'log2'	1	2	125	1
'log2'	1	3	25	1
'log2'	1	3	50	3
'log2'	1	3	75	1
'log2'	1	3	100	4
'log2'	1	3	125	4
'log2'	1	3	150	6
'log2'	2	2	25	2
'log2'	2	2	50	3
'log2'	2	2	75	2
'log2'	2	2	125	2
'log2'	2	3	25	1
'log2'	2	3	50	1
'log2'	2	3	100	1
'log2'	2	3	125	2
'log2'	2	3	150	2
'sqrt'	1	2	25	1
'sqrt'	1	2	50	6
'sqrt'	1	2	75	2
'sqrt'	1	2	100	2
'sqrt'	1	2	125	1
'sqrt'	1	2	150	5
'sqrt'	1	3	25	3
'sqrt'	1	3	50	1
'sqrt'	1	3	75	5
'sqrt'	1	3	100	5
'sqrt'	1	3	125	4
'sqrt'	1	3	150	2
'sqrt'	2	2	50	1
'sqrt'	2	2	75	1
'sqrt'	2	2	100	2
'sqrt'	2	2	150	4
'sqrt'	2	3	25	2
'sqrt'	2	3	50	4
'sqrt'	2	3	75	1
'sqrt'	2	3	125	3
'sqrt'	2	3	150	3

Table S4. Performance of newly trained machine learning models with pre-defined radiomics features

Training: Centre 1, testing: Centre 1						
Model	Training set - AUC	Test set - AUC	Test set - SN	Test set - SP	Test set - PPV	Test set - NPV
Random forest	0.766 (0.087)	0.726 (0.102)	0.731 (0.126)	0.642 (0.196)	0.824 (0.084)	0.542 (0.127)

Support vector machine	0.810 (0.057)	0.800 (0.093)	0.753 (0.134)	0.743 (0.186)	0.873 (0.083)	0.610 (0.146)
Logistic regression	0.819 (0.050)	0.809 (0.095)	0.768 (0.133)	0.750 (0.197)	0.879 (0.085)	0.631 (0.155)
Majority vote	/	0.785 (0.093)	0.769 (0.114)	0.672 (0.208)	0.845 (0.085)	0.590 (0.129)
Training: Centre 2, testing: Centre 2						
Model	Training set - AUC	Test set - AUC	Test set - SN	Test set - SP	Test set - PPV	Test set - NPV
Random forest	0.508 (0.119)	0.431 (0.138)	0.252 (0.177)	0.659 (0.126)	0.192 (0.132)	0.724 (0.057)
Support vector machine	0.529 (0.117)	0.449 (0.128)	0.354 (0.199)	0.583 (0.173)	0.233 (0.134)	0.726 (0.071)
Logistic regression	0.551 (0.119)	0.493 (0.133)	0.422 (0.209)	0.571 (0.142)	0.250 (0.126)	0.750 (0.065)
Majority vote	/	0.434 (0.127)	0.270 (0.194)	0.667 (0.118)	0.207 (0.137)	0.732 (0.064)
Training: Centre 1, testing: Centre 2						
Model	Training set - AUC	Test set - AUC	Test set - SN	Test set - SP	Test set - PPV	Test set - NPV
Random forest	0.714	0.561	0.625	0.633	0.357	0.838
Support vector machine	0.806	0.630	0.563	0.694	0.375	0.829
Logistic regression	0.819	0.630	0.563	0.633	0.333	0.816
Majority vote	/	0.614	0.563	0.653	0.346	0.821
Training: Centre 2, testing: Centre 1						
Model	Training set - AUC	Test set - AUC	Test set - SN	Test set - SP	Test set - PPV	Test set - NPV
Random forest	0.499	0.519	0.286	0.750	0.706	0.333
Support vector machine	0.543	0.699	0.643	0.650	0.794	0.464
Logistic regression	0.542	0.765	0.738	0.800	0.886	0.593
Majority vote	/	0.636	0.310	0.750	0.722	0.341
Training: Centre 1 + Centre 2, testing: Centre 1 + Centre 2						
Model	Training set - AUC	Test set - AUC	Test set - SN	Test set - SP	Test set - PPV	Test set - NPV
Random forest	0.622 (0.062)	0.599 (0.068)	0.531 (0.114)	0.625 (0.120)	0.555 (0.081)	0.610 (0.066)
Support vector machine	0.696 (0.046)	0.722 (0.075)	0.667 (0.135)	0.677 (0.101)	0.642 (0.073)	0.713 (0.078)
Logistic regression	0.706 (0.041)	0.728 (0.074)	0.683 (0.118)	0.680 (0.099)	0.650 (0.074)	0.721 (0.073)
Majority vote	/	0.669 (0.062)	0.594 (0.105)	0.659 (0.106)	0.605 (0.072)	0.657 (0.057)

Section 1: Training and test in Centre 1; **Section 2:** Training and test in Centre 2; **Section 3:** Training in Centre 1 and test in Centre 2; **Section 4:** Training in Centre 2 and test in Centre 1; **Section 5**: Data from Centre 1 and Centre 2 used for both training and testing. Mean performance and standard deviation in brackets.

Table S5. Comparisons between newly trained machine learning models

Training: Centre 1, testing: Centre 1 - Wilcoxon signed-rank test p values			
	Logistic regression	Majority vote	Random forest
Majority vote	< 0.001 ***	/	/
Random forest	< 0.001 ***	< 0.001 ***	/
Support vector machine	0.055	< 0.001 ***	< 0.001 ***
**Training: Centre 2, testing: Centre 2 - Wilcoxon signed-rank test p values**			
	Logistic regression	Majority vote	Random forest
Majority vote	< 0.001 ***	/	/
Random forest	0.001 **	0.612	/
Support vector machine	< 0.001 ***	0.394	0.461
**Training: Centre 1, testing: Centre 2 - De Long test p values**			
	Logistic regression	Majority vote	Random forest
Majority vote	0.349	/	/
Random forest	0.349	0.349	/
Support vector machine	0.914	0.349	0.349
**Training: Centre 2, testing: Centre 1 - De Long test p values**			
	Logistic regression	Majority vote	Random forest
Majority vote	<0.001 ***	/	/
Random forest	<0.001 ***	<0.001 ***	/
Support vector machine	0.621	<0.001 ***	<0.001 ***
**Training: Centre 1 + Centre 2, testing: Centre 1 + Centre 2 - Wilcoxon signed-rank test p values**			
	Logistic regression	Majority vote	Random forest
Majority vote	< 0.001 ***	/	/
Random forest	< 0.001 ***	< 0.001 ***	/
Support vector machine	< 0.001 ***	< 0.001 ***	< 0.001 ***
**New radiomics signature - Wilcoxon signed-rank test p values**			
	Logistic regression	Majority vote	Random forest
Majority vote	< 0.001 ***	/	/
Random forest	< 0.001 ***	< 0.001 ***	/
Support vector machine	0.005 **	< 0.001 ***	< 0.001 ***

Correlation matrices showing p values for Wilcoxon-signed rank tests (section 1, 2, 5) and De Long test (section 3 and 4) between all models' performances (for each classifier); * p <0.05, ** p < 0.01, *** p < 0.001.

Table S6. METRICS score

Items/Conditions	Definitions	Weights	
Study Design			
Item#1	Adherence to radiomics and/or machine learning-specific checklists or guidelines	0.0368	yes
Item#2	Eligibility criteria that describe a representative study population	0.0735	yes
Item#3	High-quality reference standard with a clear definition	0.0919	yes
Imaging Data			
Item#4	Multi-center	0.0438	yes
Item#5	Clinical translatability of the imaging data source for radiomics analysis	0.0292	yes

Item#6	Imaging protocol with acquisition parameters	0.0438	yes
Item#7	The interval between imaging used and reference standard	0.0292	no
Segmentation C			
Condition#1	Does the study include segmentation?		yes
Condition#2	Does the study include fully automated segmentation?		no
Item#8	Transparent description of segmentation methodology	0.0337	yes
Item#9	Formal evaluation of fully automated segmentation C	0.0225	n/a
Item#10	Test set segmentation masks produced by a single reader or automated tool	0.0112	no
Image Processing and Feature Extraction			
Condition#3	Does the study include hand-crafted feature extraction?		yes
Item#11	Appropriate use of image preprocessing techniques with transparent description	0.0622	yes
Item#12	Use of standardized feature extraction software C	0.0311	yes
Item#13	Transparent reporting of feature extraction parameters, otherwise providing a default configuration statement	0.0415	yes
Feature Processing			
Condition#4	Does the study include tabular data?		yes
Condition#5	Does the study include end-to-end deep learning?		no
Item#14	Removal of non-robust features C	0.0200	no
Item#15	Removal of redundant features C	0.0200	yes
Item#16	Appropriateness of dimensionality compared to data size C	0.0300	yes
Item#17	Robustness assessment of end-to-end deep learning pipelines C	0.0200	n/a
Preparation for Modeling			
Item#18	Proper data partitioning process	0.0599	yes
Item#19	Handling of confounding factors	0.0300	no
Metrics and Comparison			
Item#20	Use of appropriate performance evaluation metrics for task	0.0352	yes
Item#21	Consideration of uncertainty	0.0234	yes
Item#22	Calibration assessment	0.0176	no
Item#23	Use of uni-parametric imaging or proof of its inferiority	0.0117	yes
Item#24	Comparison with a non-radiomic approach or proof of added clinical value	0.0293	yes
Item#25	Comparison with simple or classical statistical models	0.0176	no
Testing			
Item#26	Internal testing	0.0375	yes
Item#27	External testing	0.0749	yes
Open Science			
Item#28	Data availability	0.0075	no

Item#29	Code availability	0.0075	no
Item#30	Model availability	0.0075	yes
Total METRICS score:			85.3%
Quality category:			Excellent

Table S7. CLEAR checklist

Section	No.	Item	Yes	No	n/a	Page
Title						
	1	Relevant title, specifying the radiomic methodology	x	☐	☐	1
Abstract						
	2	Structured summary with relevant information	x	☐	☐	1
Keywords						
	3	Relevant keywords for radiomics	x	☐	☐	2
Introduction						
	4	Scientific or clinical background	x	☐	☐	3
	5	Rationale for using a radiomic approach	x	☐	☐	3
	6	Study objective(s)	x	☐	☐	3
Method						
Study Design	7	Adherence to guidelines or checklists (e.g., CLEAR checklist)	x	☐	☐	7
	8	Ethical details (e.g., approval, consent, data protection)	X	☐	☐	5
	9	Sample size calculation	☐	X	☐	
	10	Study nature (e.g., retrospective, prospective)	X	☐	☐	5
	11	Eligibility criteria	x	☐	☐	5
	12	Flowchart for technical pipeline	X	☐	☐	6
Data	13	Data source (e.g., private, public)	x	☐	☐	5
	14	Data overlap	x	☐	☐	6
	15	Data split methodology	X	☐	☐	6
	16	Imaging protocol (i.e., image acquisition and processing)	x	☐	☐	5
	17	Definition of non-radiomic predictor variables	☐	☐	X	
	18	Definition of the reference standard (i.e., outcome variable)	x	☐	☐	5
Segmentation	19	Segmentation strategy	x	☐	☐	5

	20	Details of operators performing segmentation	x	☐	☐	5
Pre-processing	21	Image pre-processing details	x	☐	☐	5
	22	Resampling method and its parameters	x	☐	☐	5
	23	Discretization method and its parameters	x	☐	☐	5
	24	Image types (e.g., original, filtered, transformed)	x	☐	☐	6
Feature extraction	25	Feature extraction method	x	☐	☐	6
	26	Feature classes	x	☐	☐	6
	27	Number of features	x	☐	☐	5
	28	Default configuration statement for remaining parameters	x	☐	☐	6
Data preparation	29	Handling of missing data	☐	☐	X	
	30	Details of class imbalance	x	☐	☐	5
	31	Details of segmentation reliability analysis	☐	X	☐	
	32	Feature scaling details (e.g., normalization, standardization)	x	☐	☐	6
	33	Dimension reduction details	x	☐	☐	6
Modeling	34	Algorithm details	x	☐	☐	6
	35	Training and tuning details	x	☐	☐	7
	36	Handling of confounders	☐	x	☐	
	37	Model selection strategy	x	☐	☐	7
Evaluation	38	Testing technique (e.g., internal, external)	x	☐	☐	6
	39	Performance metrics and rationale for choosing	x	☐	☐	7
	40	Uncertainty evaluation and measures (e.g., confidence intervals)	x	☐	☐	7
	41	Statistical performance comparison (e.g., DeLong's test)	X	☐	☐	7
	42	Comparison with non-radiomic and combined methods	x	☐	☐	7
	43	Interpretability and explainability methods	☐	x	☐	
Results						
	44	Baseline demographic and clinical characteristics	x	☐	☐	8
	45	Flowchart for eligibility criteria	X	☐	☐	5
	46	Feature statistics (e.g., reproducibility, feature selection)	☐	☐	X	

	47	Model performance evaluation	x	☐	☐	8
	48	Comparison with non-radiomic and combined approaches	x	☐	☐	8
Discussion						
	49	Overview of important findings	x	☐	☐	9
	50	Previous works with differences from the current study	x	☐	☐	9
	51	Practical implications	x	☐	☐	10
	52	Strengths and limitations (e.g., bias and generalizability issues)	x	☐	☐	10
Open Science						
Data availability	53	Sharing images along with segmentation data [n/e]	☐	X	☐	
	54	Sharing radiomic feature data	☐	X	☐	
Code availability	55	Sharing pre-processing scripts or settings	x	☐	☐	6
	56	Sharing source code for modeling	☐	X	☐	
Model availability	57	Sharing final model files	☐	X	☐	
	58	Sharing a ready-to-use system [n/e]	☐	x	☐	

Figure S1. Features used in the new bi-centric signature

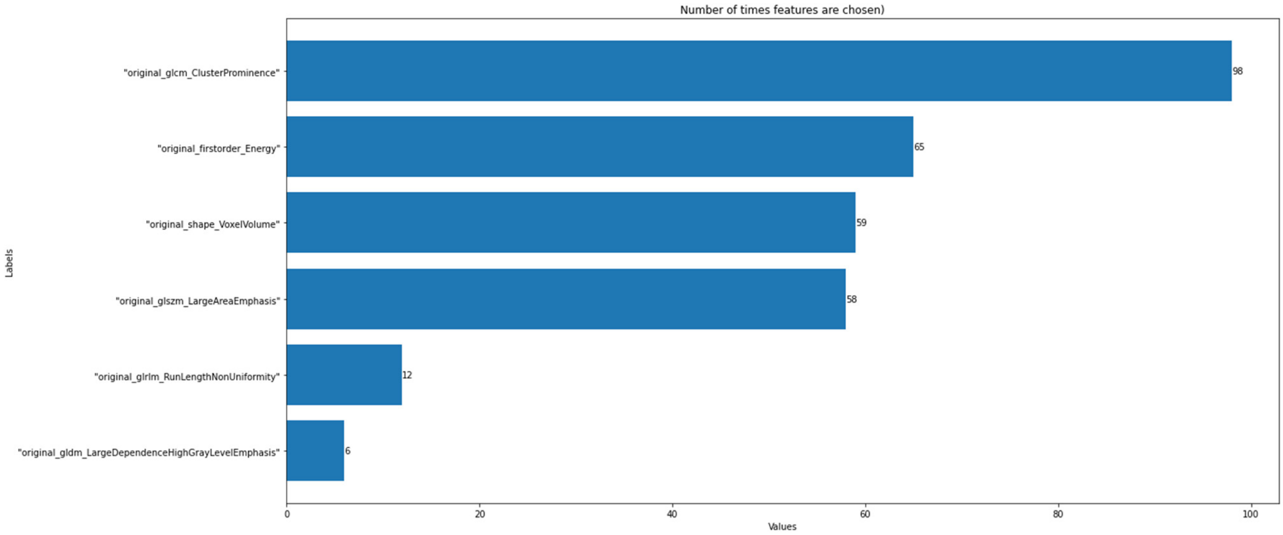

Supplement: Supplementary file 1 [file cancers-16-04103-s001.zip › cancers-3318305-supplementary.pdf]
